# Phase II study of oxaliplatin combined with S-1 and leucovorin (SOL) for Chinese patients with metastatic colorectal cancer

**DOI:** 10.1186/s40880-015-0061-3

**Published:** 2016-01-06

**Authors:** Zhi-Qiang Wang, Dong-Sheng Zhang, Nong Xu, De-Yun Luo, Yan-Hong Deng, Feng-Hua Wang, Hui-Yan Luo, Miao-Zhen Qiu, Yu-Hong Li, Rui-Hua Xu

**Affiliations:** Sun Yat-sen University Cancer Center, State Key Laboratory of Oncology in South China, Collaborative Innovation Center of Cancer Medicine, 510060 Guangzhou, Guangdong P. R. China; Department of Medical Oncology, Sun Yat-sen University Cancer Center, 651 Dongfeng Road East, 510060 Guangzhou, Guangdong P. R. China; Department of Medical Oncology, The First Affiliated Hospital of College of Medicine, Zhejiang University, 310003 Hangzhou, Zhejiang P. R. China; Department of Medical Oncology, West China Hospital of Sichuan University, 610041 Chengdu, Sichuan P. R. China; Department of Medical Oncology, The Sixth Affiliated Hospital of Sun Yat-sen University, 510655 Guangzhou, Guangdong P. R. China

**Keywords:** Colorectal cancer, Oxaliplatin, S-1, Leucovorin

## Abstract

**Background:**

Fluoropyrimidine and oxaliplatin are widely used for patients with colorectal cancer. This phase II study was conducted to evaluate the efficacy and safety of the combination of S-1, oxaliplatin, and leucovorin (SOL) in the treatment of Chinese patients with metastatic colorectal cancer (mCRC).

**Methods:**

Eligible patients with untreated mCRC from four hospitals in China received intravenous oxaliplatin (85 mg/m^2^) on day 1, oral S-1 twice daily (80–120 mg per day) on day 1–7, and leucovorin twice daily (50 mg per day) simultaneously with S-1, every 2 weeks.

**Results and discussion:**

Forty patients were enrolled in our study. In total, 296 cycles of SOL were administered. The overall response rate was 50.0%. At a median follow-up of 27 months, progression-free survival and overall survival were 7.0 months (95% confidence interval [CI] 6.0–10.6 months) and 22.2 months (95% CI 15.1–29.3 months), respectively. The most common grade 3/4 non-hematological adverse events were diarrhea (*n* = 8, 20.0%), nausea (*n* = 3, 7.5%), and vomiting (*n* = 3, 7.5%). The most common grade 3/4 hematological toxicities were thrombocytopenia (*n* = 3, 7.5%), neutropenia (*n* = 1, 2.5%), and abnormal alanine transaminase/aspartate transaminase levels (*n* = 1, 2.5%). There was one treatment-related death.

**Conclusions:**

The data indicate that the SOL regimen is effective and moderately tolerated in Chinese patients with mCRC.

Trial registration: Clinical trial information: ChiCTR-TNRC-100000838

## Background

In 2010, colorectal cancer was the fifth most common cancer in men and the third most common cancer in women in China [1]. At present, the combination of oxaliplatin with infusional 5-fluorouracil (5-FU) and leucovorin (known as the FOLFOX regimen) or the combination of irinotecan with infusional 5-FU and leucovorin (known as the FOLFIRI regimen) are considered the standard first-line chemotherapeutic regimens for the treatment of patients with metastatic colorectal cancer (mCRC) [2–4]. However, the FOLFOX and FOLFIRI regimens are less desirable because 5-FU must be continuously infused via vascular access. To overcome the inconvenience of continuous infusion of 5-FU, oral fluoropyrimidines, such as capecitabine, have been substituted for infusional 5-FU/leucovorin. Previous studies have shown that capecitabine plus oxaliplatin (CapeOX) was not inferior to the FOLFOX regimen [5].

S-1, a novel dihydropyrimidine dehydrogenase-inhibitory oral fluoropyrimidine, has been prescribed widely for patients with gastric cancer in eastern Asia [6–8]. In phase II studies, S-1, as a single agent, resulted in an overall response rate (ORR) of 19%–40% after first-line treatment of mCRC [9–11]. Fluoropyrimidines are integral components of the treatment regimen for patients with mCRC. A meta-analysis by Thirion et al. [12] demonstrated that 5-FU plus leucovorin improved the ORR and prolonged overall survival (OS) of patients with mCRC compared with single-agent 5-FU. A Japanese study [13] indicated that S-1 combined with leucovorin (2 weeks on and 2 weeks off) showed good efficacy in the first-line treatment of mCRC; however, patients in this study who received S-1 and leucovorin (2 weeks on and 2 weeks off) had a high occurrence rate of diarrhea, anorexia, and stomatitis. The result of a phase II study showed that patients who received S-1 plus leucovorin (1 week on and 1 week off) had a high ORR and a low occurrence rate of adverse reactions [14].

In an effort to achieve higher efficacy than S-1 and leucovorin (1 week on and 1 week off) alone and evaluate the safety, we carried out a phase II clinical trial with a regimen of oxaliplatin combined with S-1 and leucovorin (SOL; 1 week on and 1 week off) in previously untreated Chinese patients with mCRC.

## Patients and methods

### Eligibility

This study was approved by the ethics committee of Sun Yat-sen University Cancer Center. The reference number is YP2010031. Eligible patients met the following criteria: presence of unresectable, metastatic, and histologically confirmed colorectal adenocarcinoma; adequate oral intake; older than 20 years; Eastern Cooperative Oncology Group performance status of 0–1; estimated life expectancy of more than 3 months; no prior chemotherapy or only fluoropyrimidine adjuvant chemotherapy that was administered more than 6 months before enrollment, or oxaliplatin-based adjuvant chemotherapy that was administered more than 1 year before enrollment; and at least one measurable disease by Response Evaluation Criteria in Solid Tumors (RECIST 1.0). Also, patients had to have adequate hematological, renal, and hepatic functions, as defined by a white blood cell count of 4.0 × 10^9^–12.0 × 10^9^/L, absolute granulocyte count ≥2.0 × 10^9^/L, platelet count ≥100 × 10^9^/L, hemoglobin level ≥90 g/L, serum creatinine level less than the upper limit of normal, serum bilirubin level less than 1.5 times the upper limit of normal, serum aspartate aminotransferase, alanine aminotransferase, and alkaline phosphatase levels no more than 2.5 times the upper limits of normal (for patients with liver metastasis, less than five times). Finally, they had to provide written informed consent.

Patients were excluded from this study if they had a history of serious hypersensitivity to fluoropyrimidine, oxaliplatin, or leucovorin; an active infection; serious concomitant diseases or conditions (such as intestinal obstruction, bleeding, pulmonary fibrosis, heart failure, renal failure, or liver failure); severe ascites or pleural effusion; extensive bone metastasis; brain metastasis or symptoms of brain metastasis; diarrhea; or another synchronous cancer. We also excluded patients who participated in another clinical study less than 4 weeks previously; women who were pregnant, nursing, possibly pregnant, or planning to become pregnant; and men intending to conceive children.

## Treatment plan

Oxaliplatin 85 mg/m^2^ mixed with 500 mL of dextrose solution was administered intravenously over 3 h on day 1. The dosage of S-1 was determined according to the patient’s body surface area (BSA) as follows: BSA <1.25 m^2^, 40 mg; 1.25 m^2^ ≤ BSA < 1.50 m^2^, 50 mg; BSA ≥1.50 m^2^, 60 mg. Leucovorin was given at a fixed dose of 25 mg. S-1 and leucovorin were administered together orally, twice daily from day 1 to 7, followed by a 7-day rest period. The treatment was repeated every 2 weeks until progression of the disease, the development of unacceptable toxicity, or patient withdrawal from the study.

## Dose modifications

The doses of oxaliplatin and S-1 were adjusted when severe toxicities occurred. When both agents caused toxicities, the doses of both were reduced. The dose of leucovorin did not need to be reduced. Treatment was interrupted in the case of grade 2 or higher toxicity (except alopecia) and was not resumed until the toxicity had been resolved or alleviated to grade 0 or 1. The dose of oxaliplatin was reduced to 65 mg/m^2^ for related grades 3–4 toxicity (or grade 2 peripheral neuropathy). The dose of S-1 was reduced by 20 mg/day for related grade 3 toxicity. The dose of oxaliplatin was reduced to 50 mg/m^2^ if the same grade 3 toxicity occurred a second time. The dose of S-1 was reduced by 40 mg/day if the same grade 3 toxicity occurred a second time. No dose increasing was permitted. Treatment was discontinued if the same grade 3 toxicity occurred a third time or the same grade 4 toxicity occurred a second time, despite dose reduction. In addition, if the toxicity was not alleviated to grade 0 or 1 after 4 weeks, the patient was excluded from the study.

## Response and toxicity evaluation

RECIST 1.0 criteria were used to evaluate tumor response, and the National Cancer Institute-Common Toxicity Criteria for Adverse Events version 3.0 were used to assess toxicity. Tumor responses were evaluated every three cycles by three-dimensional computed tomography or magnetic resonance imaging. All complete and partial responses were assessed and confirmed no less than 4 weeks after the criteria for response were first met. After completion of the study, patients were followed up every 3 months until disease progression or death.

### Statistical analysis

The primary endpoints of this study were ORR and progression-free survival (PFS). The secondary endpoints were safety and OS. PFS was defined as time from the initiation of treatment to the first documentation of disease progression or death from any cause. OS was measured from the start of treatment to the last follow-up or death. PFS and OS were estimated based on Kaplan–Meier plots and are presented as median values with a 95% confidence interval (CI).

Simon’s optimal two-stage design was used to test the null hypothesis (80% statistical power and 5% significance), the true ORR was less than 20%, against the alternative hypothesis, the true response rate was greater than 40%. As predefined by the protocol, at least six responses were required among the first 17 assessable patients for the study to continue. In the second stage, 21 additional patients were enrolled to achieve a target sample size of 38 assessable patients. Assuming a dropout rate of 5%, 40 patients were initially required for the study.

## Results

### Patient characteristics

Between March 2010 and October 2012, 40 patients from four hospitals were enrolled. Patient characteristics are listed in Table 1. The median age was 56 years (range 21–79 years), and most patients were men (*n* = 27, 67.5%). Twenty-three (57.5%) patients had colon cancer. All patients were histologically confirmed as having adenocarcinoma. The majority (72.5%) had moderately differentiated tumors. Thirty-three patients (82.5%) had resected primary tumors. Sixteen patients (40.0%) had previously received adjuvant chemotherapy. The most common metastatic site was the liver (*n* = 24). The median number of metastatic organs was two (range 1–5). PFS, OS, and safety were assessed for all patients. Five patients were unavailable for the response analysis due to severe adverse events (*n* = 1) and withdrawing the informed consent (*n* = 4).

**Table 1:** Baseline demographic and clinical characteristics of enrolled patients with mCRC

Characteristic	No. of patients [cases (%)]
Sex	
Male	27 (67.5)
Female	13 (32.5)
ECOG PS	
0	15 (37.5)
1	25 (62.5)
Primary disease site	
Colon	23 (57.5)
Rectosigmoid colon	1 (2.5)
Rectum	16 (40.0)
Primary tumor resection	
No	7 (17.5)
Yes	33 (82.5)
Adjuvant chemotherapy	
No	24 (60.0)
Yes	16 (40.0)
Differentiation	
Well	1 (2.5)
Moderate	29 (72.5)
Poor	3 (7.5)
Unknown	7 (17.5)
Metastatic sites	
Liver	24 (60.0)
Lung	16 (40.0)
Lymph nodes	10 (25.0)
Peritoneum	8 (20.0)
Others	4 (10.0)
No. of metastatic sites	
1	14 (35.0)
2	18 (45.0)
≥3	8 (20.0)

*mCRC* metastatic colorectal cancer, *ECOG* Eastern Cooperative Oncology Group, *PS* performance status

### Response to therapy and survival

A total of 296 treatment cycles were delivered to patients. The median number of treatment cycles was nine (range 1–18). No patient had a complete response, but 20 patients had partial responses, 12 had stable disease, and three had progressive disease. The ORR was 50.0% (95% CI 33.8%–66.2%). Tumor response rates are listed in Table 2.

**Table 2:** The responses of 40 Chinese patients with mCRC to the SOL regimen

Response	No. of patients [cases (%)]
Complete response	0
Partial response	20 (50.0)
Stable disease	12 (30.0)
Disease control	32 (80.0)
Progression	3 (7.5)
Not evaluable	5 (12.5)
Overall response^a^	20 (50.0)

*SOL* the combination of S-1, oxaliplatin, and leucovorin regimen. Other abbreviation as in Table 1

^a^95% confidence interval of overall response rate is 33.8%–66.2%

**Fig. 1:**
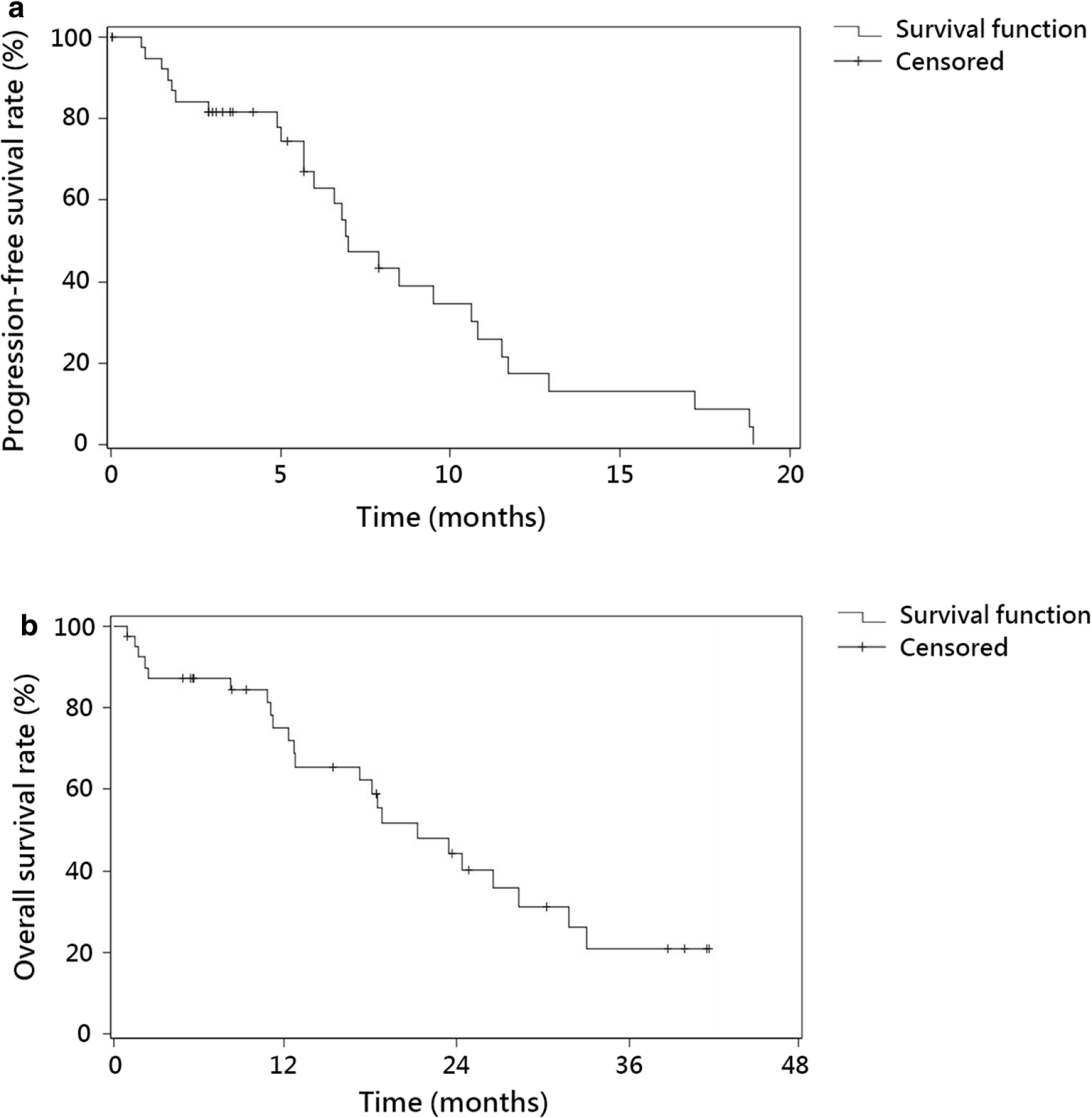
Kaplan-Meier curves of progression-free survival and overall survival for 40 Chinese patients with metastatic colorectal cancer. **a** The estimated progression-free survival was 7.0 months (95% confidence interval [CI] 6.0–10.6 months). **b** The estimated overall survival was 22.2 months (95% CI 15.1–29.3 months)

The median follow-up time was 27 months (range 10–48 months). The median PFS was 7.0 months (95% CI 6.0–10.6 months; Fig. 1a). The median OS was 22.2 months (95% CI 15.1–29.3 months; Fig. 1b).

## Safety

Safety was assessed in 40 patients based on a total of 296 treatment cycles. Adverse events are listed in Table 3. Diarrhea, which was experienced by 20.0% of the patients, was the most common grade 3/4 adverse event. Neuropathy, which was experienced by 12.5% of the patients, was the second most common grade 3/4 toxicity. Thrombocytopenia, nausea, and vomiting, which were experienced by 7.5% of the patients, were the third most common grade 3/4 toxicities. Other non-hematologic toxic effects were usually mild (mostly grade 1/2) and manageable.

**Table 3:** The adverse events in the 40 Chinese patients with mCRC treated with the SOL regimen

Event	NCI-CTCAE grade, version 3.0 [cases (%)]		
	1	2	3
Leukopenia	7 (17.5)	7 (17.5)	0
Neutropenia	6 (15.0)	4 (10.0)	1 (2.5)
Anemia	3 (7.5)	3 (7.5)	0
Thrombocytopenia	5 (12.5)	7 (17.5)	3 (7.5)
Anorexia	19 (47.5)	8 (20.0)	0
Nausea	16 (40.0)	6 (15.0)	3 (7.5)
Vomiting	6 (15.0)	6 (15.0)	3 (7.5)
Diarrhea	5 (12.5)	9 (22.5)	8 (20.0)
Stomatitis	4 (10.0)	2 (5.0)	1 (2.5)
Constipation	4 (10.0)	0	0
Fatigue	18 (45.0)	5 (12.5)	1 (2.5)
Alopecia	29 (72.5)	6 (15.0)	0
Pigmentation	5 (12.5)	14 (35.0)	0
Hand-foot syndrome	14 (35.0)	2 (5.0)	1 (2.5)
Neuropathy	21 (52.5)	12 (30.0)	5 (12.5)
Abnormal ALT/AST	7 (17.5)	3 (7.5)	1 (2.5)

*NCI-CTCAE* National Cancer Institute-Common Terminology Criteria for Adverse Events, *ALT/AST* alanine transaminase/aspartate transaminase. Other abbreviations as in Tables 1 and 2

In total, 160 (54.0%) treatment cycles were delayed. Sixty (21.0%) cycles required a dose reduction of oxaliplatin. The causes of dose reduction of oxaliplatin included neuropathy, thrombocytopenia, and diarrhea. S-1 was reduced in 56 (19.0%) cycles because patients experienced diarrhea, hand-foot syndrome, thrombocytopenia, and stomatitis. During the first cycle of treatment, one patient died because of grade 3 diarrhea and febrile neutropenia with lung infection.

## Discussion

This phase II study evaluated the efficacy and safety of SOL in the first-line treatment of Chinese patients with mCRC. The ORR was 50.0%, median PFS was 7.0 months, and median OS was 22.2 months. The common adverse events were diarrhea, neuropathy, thrombocytopenia, nausea, and vomiting.

FOLFOX and CapeOX are the standard regimens in the first-line treatment of patients with mCRC. In previous studies, the ORR of FOLFOX or CapeOx was determined to be 37%–55%, the median PFS was 6–9.5 months, and the median OS was 16.2–20.8 months with these first-line treatments [2–5, 15–21]. In recent years, capecitabine and 5-FU can be substituted by S-1 in the treatment of mCRC patients in Japan [22]. A phase II study evaluated the efficacy of the combination of oxaliplatin and S-1 (SOX) in the first-line treatment of mCRC. In this study, the ORR was 54%, the median time to progression (TTP) was 8.5 months, and the median OS was 27.2 months [23]. A phase III study also evaluated the efficacy of SOX and CapeOX in the first-line treatment of mCRC. In this study, the ORR was 47% and 36%, the median PFS was 8.5 and 6.7 months, and the median OS was 21.2 and 20.5 months, respectively [24]. Collectively, the efficacy data from all the previous studies are similar to the efficacy data of the SOL regimen in our study.

Mechanistically, the anti-tumor activity of 5-FU is considered to result from the formation of a ternary complex of 5-fluoro-2′-deoxyuridine-5′-monophosphate (metabolite of 5-FU), thymidylate synthase, and 5,10-methylenete terahydrofolate (metabolite of leucovorin). This complex inhibits thymidylate synthase, thereby blocking DNA synthesis [25]. In a phase II study, the anti-tumor activity of S-1 was enhanced by oral leucovorin due to this mechanism [13]. This study showed an ORR of 57%, a median TTP of 6.7 months, and a median OS of 24.3 months in patients with mCRC treated with the combination of S-1 and leucovorin (2 weeks on and 2 weeks off). The most common grade 3/4 toxicities were diarrhea (32%), anorexia (21%), mucositis (20%), and neutropenia (14%). The toxicities first occurred during the 2nd week of treatment. Given these data, the combination of S-1 and leucovorin (1 week on and 1 week off) may have the same efficacy and lower toxicity. The results of a recent phase II study showed that S-1 and leucovorin (1 week on and 1 week off) produced an ORR of 53.5%, a median TTP of 6.5 months, and a median OS of 24.3 months in the first-line treatment of Mcrc [14]. It showed that the efficacy of S-1 and leucovorin (1 week on and 1 week off) was similar to that of FOLFOX or CapeOX [2–5, 11–13, 15–18]. The most common grade 3/4 toxicities of S-1 and leucovorin (1 week on and 1 week off) were diarrhea (8.3%), anorexia (2.8%), mucositis (8.3%), and neutropenia (9.4%), which were less common than the toxicities observed during the treatment with the S-1 and leucovorin (2 weeks on and 2 weeks off) regimen [13, 14].

We hypothesized that the efficacy might be improved when S-1 and leucovorin were combined with oxaliplatin. Therefore, the current study was designed to test the efficacy and safety of the SOL regimen for the first-line treatment of Chinese patients with mCRC.

In our study, diarrhea was the most common grade 3/4 toxicity, which occurred in eight of 40 patients (20.0%). The high occurrence rate of diarrhea was unexpected. One patient died because of grade 3 diarrhea and febrile neutropenia with lung infection after the first cycle. Previous data showed that the occurrence rate of grade 3/4 diarrhea was about 10% when patients received S-1 and leucovorin or SOL (1 week on and 1 week off) [14]. The reason for the high occurrence rate of diarrhea in our study was unknown. It may be related to the small sample size or the expression of the fluoropyrimidine metabolic enzymes, such as dihydropyrimidine dehydrogenase. Although peripheral neuropathy was commonly observed (95.0%), most cases were grade 1 and grade 2. The occurrence rates of other common grade 3/4 toxicities, such as neutropenia, anorexia, and fatigue, were lower than those observed in a Japanese study [26]. In our study, approximately 54.0% of the treatment cycles had to be delayed due to the toxicities. The dose of oxaliplatin was reduced in 21.0% of the cycles, and the dose of S-1was reduced in 19.0% of the cycles due to diarrhea and other toxicities.

A Japanese randomized phase II study compared SOL with FOLFOX in patients with untreated mCRC [26]. In this study, the median PFS was 9.6 months in the SOL group and 6.9 months in the FOLFOX group (hazard ratio [HR], 0.83; 95% CI 0.49–1.40), and the median OS was 28.5 and 25.9 months, respectively (HR, 0.91; 95% CI 0.55–1.49). The efficacy shown in our study seemed to be worse than that in the Japanese study. This may be due to the limited sample size, the high rate of toxicities, and low dose density in our study.

In conclusion, the SOL regimen seems to be an effective, moderately tolerated, and convenient therapeutic strategy for the first-line treatment of Chinese patients with mCRC. A phase III study is necessary to validate the clinical outcomes of the SOL regimen.
